# Density Functional Theory-Based Approaches to Improving Hydrogen Storage in Graphene-Based Materials

**DOI:** 10.3390/molecules29020436

**Published:** 2024-01-16

**Authors:** Heriberto Cruz-Martínez, Brenda García-Hilerio, Fernando Montejo-Alvaro, Amado Gazga-Villalobos, Hugo Rojas-Chávez, Elvia P. Sánchez-Rodríguez

**Affiliations:** 1 Tecnológico Nacional de México, Instituto Tecnológico del Valle de Etla, Abasolo S/N, Barrio del Agua Buena, Santiago Suchilquitongo, Oaxaca 68230, Mexico; heri1234@hotmail.com (H.C.-M.); brenda.hilerio@itvalletla.edu.mx (B.G.-H.); moaf1217@gmail.com (F.M.-A.); gazgavillalobos@gmail.com (A.G.-V.); 2 Tecnológico Nacional de México, Instituto Tecnológico de Tláhuac II, Camino Real 625, Tláhuac, Ciudad de México 13550, Mexico; hugo.rc@tlahuac2.tecnm.mx; 3 School of Engineering and Sciences, Tecnologico de Monterrey, Atizapan de Zaragoza 52926, Mexico

**Keywords:** decorated graphene, defective graphene, doped graphene, decorated-doped graphene DFT calculations

## Abstract

Various technologies have been developed for the safe and efficient storage of hydrogen. Hydrogen storage in its solid form is an attractive option to overcome challenges such as storage and cost. Specifically, hydrogen storage in carbon-based structures is a good solution. To date, numerous theoretical studies have explored hydrogen storage in different carbon structures. Consequently, in this review, density functional theory (DFT) studies on hydrogen storage in graphene-based structures are examined in detail. Different modifications of graphene structures to improve their hydrogen storage properties are comprehensively reviewed. To date, various modified graphene structures, such as decorated graphene, doped graphene, graphene with vacancies, graphene with vacancies-doping, as well as decorated-doped graphene, have been explored to modify the reactivity of pristine graphene. Most of these modified graphene structures are good candidates for hydrogen storage. The DFT-based theoretical studies analyzed in this review should motivate experimental groups to experimentally validate the theoretical predictions as many modified graphene systems are shown to be good candidates for hydrogen storage.

## 1. Introduction

Hydrogen is gaining importance as a clean energy carrier with higher energy density than conventional fuels [1,2]. Although it is the most abundant element in the universe [3], it is not a primary energy source available on our planet. Therefore, various technologies have been proposed that allow for the efficient and safe production, storage, and utilization of hydrogen [4,5,6]. Currently, hydrogen is obtained from a wide range of resources, such as renewable resources and fossil fuels [7,8]. Unfortunately, the element has a low density under ambient conditions. Consequently, many storage technologies have been developed for storing it with a high density [9,10]. Diverse electrochemical systems with extremely high efficiencies have been proposed to obtain clean electrical energy from hydrogen [11,12,13].

The existing hydrogen storage technologies are based on liquefaction or compression or a combination of the two. However, the liquefaction and pressurization of hydrogen are not economically viable alternatives for hydrogen storage [14,15]. Hence, hydrogen storage in materials is considered a good storage option [14] because some of the explored materials provide H_2_ storage capacities like or better than the requirements prescribed by the U.S. Department of Energy (DOE) [16,17]. Therefore, in recent years, numerous materials have been explored to store hydrogen [18,19,20,21,22,23]. Among them, carbon-based materials are of high importance because of their suitable properties, such as high specific surface area, low density, and high thermal as well as chemical stability, making them promising materials for hydrogen storage [24,25].

Currently, at the molecular level, owing to advances in density functional theory (DFT)-based methods and computer equipment, novel materials with good performances have been proposed for hydrogen technologies [26,27,28,29,30]. The DFT-based approach has gained greater importance as it maintains a good balance between computational time and accuracy in terms of agreement with the experimental results [31,32,33].

**Figure 1:**
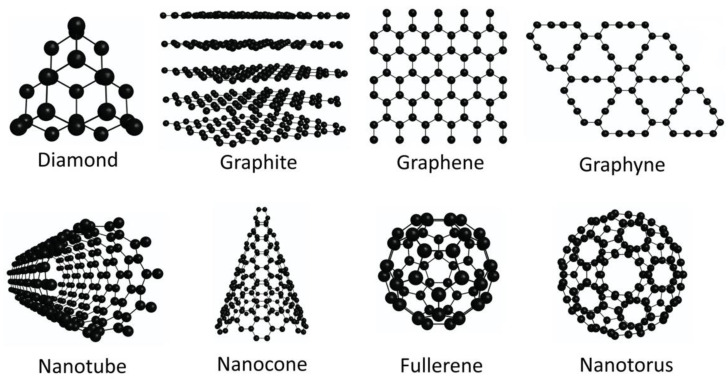
Carbon structures used for hydrogen storage.

To date, numerous carbon structures (e.g., graphene, graphite, graphene, nanotube, nanocone, fullerene, nanotorus) have been explored for hydrogen storage at the DFT level, and promising results have been achieved [34,35,36,37,38,39,40,41]. These structures are shown in Figure 1. Although carbon structures are good candidates for hydrogen storage, pristine carbon structures have limited reactivity for hydrogen storage [42,43]. Therefore, to improve the hydrogen storage properties of these structures, diverse approaches such as defect engineering and surface functionalization have been implemented. These strategies allow us to improve hydrogen storage in carbon structures such as graphene, carbon nanotubes, and fullerenes [44,45]. Consequently, the modification of carbon structures through defect engineering and surface functionalization for hydrogen storage is a relevant topic for designing novel carbon-based hydrogen storage materials. Among the structures investigated for hydrogen storage at the DFT level, graphene is the most studied structure. In this sense, there are some review articles that analyze the applicability of graphene for hydrogen storage. For instance, in 2017, some theoretical studies on the hydrogen storage properties of modified graphene were revised [45]. Recently, Singla and Jaggi reviewed the theoretical studies on graphene and its derivatives for hydrogen detection and storage applications [44]. They analyzed the effect of different dopants (i.e., alkali and alkaline earth atoms, transition metal atoms) on the properties of graphene-based structures to improve their hydrogen detection and storage capabilities [44]. These review articles show the importance of modified graphene to be used for hydrogen storage [44,45]. However, to date, there has been no detailed review article that explains in detail the modifications made to graphene structures for improving their hydrogen storage properties. Therefore, in this review, we analyze the DFT-based theoretical advances in the design of novel graphene-based hydrogen storage materials, highlighting the most popular modifications made to the graphene structure to improve the hydrogen storage properties.

## 2. Hydrogen Storage on Pristine Graphene

One of the first theoretical investigations on the use of pristine graphene to store hydrogen was reported by Ganji et al. [35]. They investigated hydrogen storage on graphene nanoflakes using the B3LYP-D3 method and demonstrated that hydrogen was adsorbed on a coronene surface with a physisorption energy of approximately −0.05 eV. In another study, H_2_ interaction on pristine graphene was investigated by using different DFT-based methods that incorporated dispersion corrections. The computed hydrogen adsorption energies on pristine graphene were less than −0.08 eV [46]. The investigated adsorption energies computed were less than the optimal hydrogen adsorption energy (−0.2 to −0.6 eV/H_2_) [47,48,49]. Therefore, to improve the hydrogen storage properties of graphene, graphene modification using methods such as defect engineering and surface functionalization is necessary. These studies also demonstrated that dispersion corrections must be included to explain the interactions of hydrogen on graphene accurately [35,46].

## 3. Hydrogen Storage on Decorated Graphene

### 3.1. Hydrogen Storage on Single-Atom Decorated Graphene

**Figure 2:**
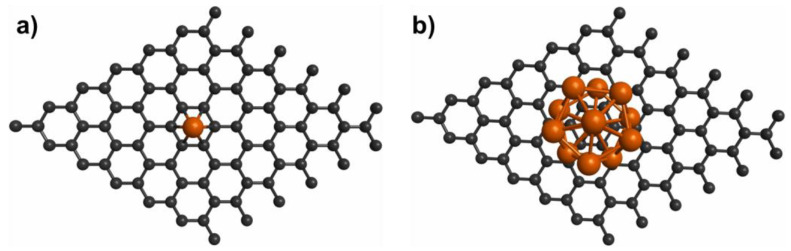
Models of decorated graphene. (**a**) Single-atom-decorated graphene, (**b**) cluster-decorated graphene.

**Figure 3:**
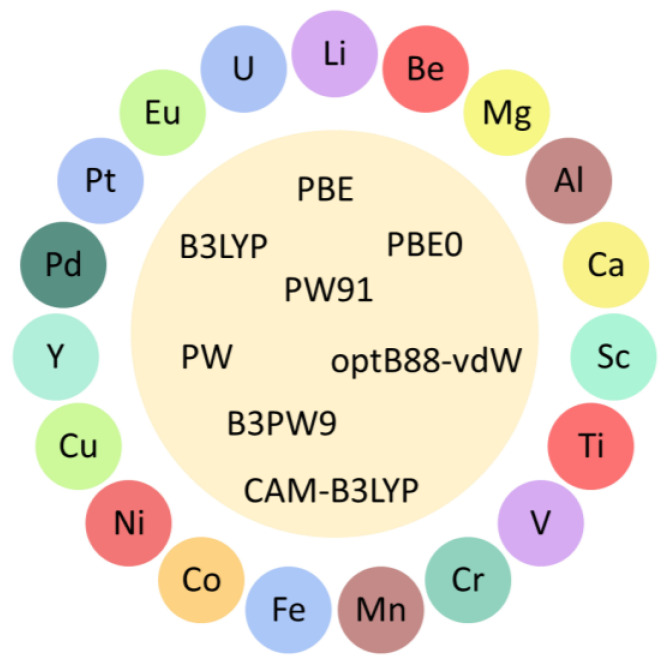
Decorative elements and computational methodologies for decorated graphene.

The use of decorated graphene is one of the strategies used to improve the hydrogen storage properties of pristine graphene. This approach involves the deposition of single-atoms (Figure 2a) or clusters (Figure 2b) on pristine graphene. Ample reports of DFT studies on hydrogen storage on decorated graphene are available in the literature [50,51,52,53,54,55,56,57,58,59,60,61,62,63,64,65,66,67,68,69,70,71,72,73,74,75,76,77,78,79,80,81,82,83,84,85,86,87,88,89,90,91,92,93]. Single-atom decoration is the commonly used strategy to decorate graphene [50,51,52,53,54,55,56,57,58,59,61,62,63,64,65,66,67,68,69,70,71,72,73,74,75,76,77,78,79,80,81,82,83,84,85,86,87,88,89,90,91,92,93]. Figure 3 shows different single-atoms that have been used to decorate graphene. The commonly used elements for decoration are Li, Ca, Ti, and Pd. Interestingly, several studies have considered dispersion corrections that substantially improve the description of the interaction between H_2_ and decorated graphene [50,52,53,56,60,62,68,72,73,74,75,81,83,85,86,90,91,93]. When the hydrogen molecule is adsorbed on graphene decorated with single-atoms, hydrogen is adsorbed on the decorating atoms as they function as active centers. Many studies showed that the adsorption energies of hydrogen on decorated graphene were higher than those of hydrogen on pristine graphene, highlighting that most single-atom-decorated graphene systems comply with the DOE requirement for hydrogen storage through physisorption. Other important parameters to consider when exploring new materials for hydrogen storage are gravimetric capacity and volumetric capacity. The 2025 targets set by the DOE are a gravimetric capacity of 5.5 wt.% and a volumetric capacity of 40 g L^−1^ for hydrogen storage systems onboard light-duty vehicles [94]. Interestingly, several of the investigated materials—decorated graphene materials such as Al [50], Ca [53,55,56,57], Li [64,65,66,67,72,88], and Ti [65,83]—possess gravimetric capacities higher than the targets set by the DOE. Thus, these studies show that single-atom-decorated graphene systems are a good strategy to store hydrogen via physisorption.

### 3.2. Hydrogen Storage in Cluster-Decorated Graphene

Another approach to decorate graphene is by using clusters (Figure 2b). Theoretical studies on cluster-decorated graphene have been reported [60,74,77]. For instance, a theoretical study examined the H_2_ interaction on Pd_n_ (n = 1–6) clusters supported on graphene using the PW91 functional [77] and reported that the H_2_ adsorption energy is close to the optimal values for hydrogen storage. In another study, hydrogen storage on Co_4_ clusters deposited on graphene was investigated using the Perdew–Burke–Ernzerhof (PBE) functional [60]; the H_2_ adsorption energy was close to the values required by the DOE. Recently, H_2_ adsorption on Li_n_ (n = 1–6) clusters supported on graphene was investigated using the PBE functional and dispersion corrections [74]. For four H_2_ molecules adsorbed on Li_6_ clusters supported on graphene, the computed adsorption energy was −0.31 eV/H_2_. Similar to single-atom-decorated graphene, clusters act as active centers in cluster-decorated graphene for hydrogen storage [60,74,77]. Thus, these studies show that the use of graphene systems decorated with clusters or atoms is a good strategy to store hydrogen via physisorption.

## 4. Hydrogen Storage on Doped Graphene

### 4.1. Hydrogen Storage on Single-Atom-Doped Graphene

**Figure 4:**
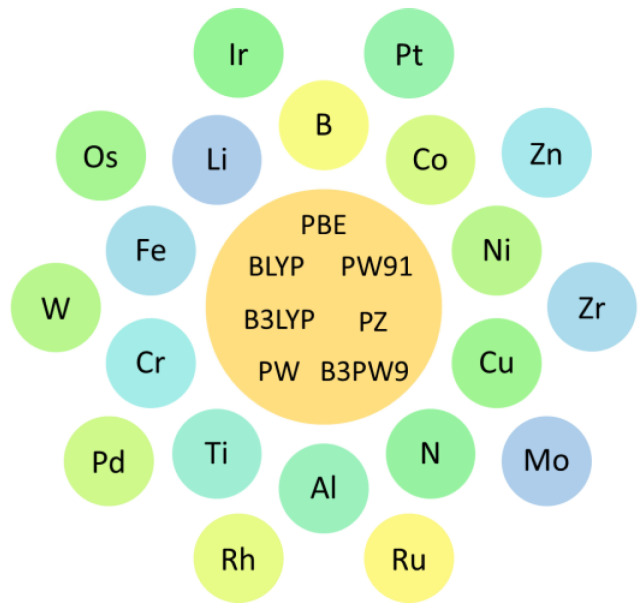
Doping elements and computational methodologies for single-atom-doped graphene.

Another route used to modify the properties of pristine graphene is through substitutional point defects such as doping. This approach substantially modifies the reactivity of pristine graphene [95,96,97,98,99,100]. At the DFT level, different types of doping have been investigated to modify the reactivity of graphene [61,62,79,101,102,103,104,105,106,107,108,109,110,111,112,113,114,115,116,117,118,119,120,121,122,123,124,125]. The commonly used route is to replace a carbon atom in the graphene structure with a dopant atom. To date, many studies have explored the development of single-atom–doped graphene for hydrogen storage [61,62,79,101,102,104,105,106,107,108,109,110,111,112,114,115,116,118,121,122,124,125]. Figure 4 shows the different single atoms used to dope graphene. The commonly used dopant atoms are N, Ti, Cu, Pd, and Pt. The PBE functional is a popular tool used to study single-atom-doped graphene for hydrogen storage. Similar to research on decorated graphene, several studies on single-atom-doped graphene for hydrogen storage adopted dispersion corrections [61,101,104,112,116,118,121,122,124,125]. Interestingly, many studies showed that the hydrogen adsorption energies on the single-atom-doped graphene fulfill the DOE requirement for hydrogen storage via physisorption. Meanwhile, the gravimetric capacities of several single-atom-doped graphene materials were close to the DOE requirement. These investigations show that the use of single-atom-doped graphene systems is a good alternative for hydrogen storage.

### 4.2. Hydrogen Storage for Different Doping Concentrations

**Figure 5:**
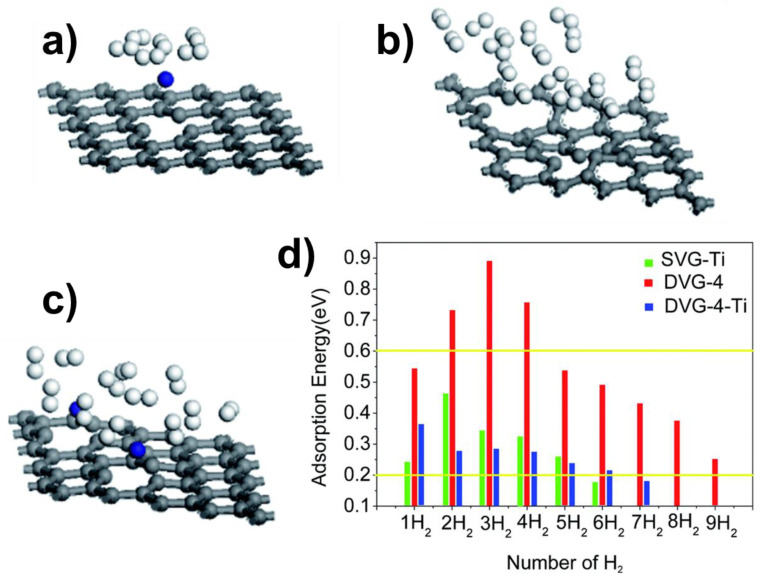
H_2_ adsorption on graphene structures. (**a**) H_2_ adsorption on Ti-doped graphene (SVG-Ti), (**b**) H_2_ adsorption on double-vacancy graphene (DVG-4), (**c**) H_2_ adsorption on Ti_2_-doped graphene (DVG-4-Ti), (**d**) hydrogen molecule adsorption energies on graphene structures. The values reported between the horizontal yellow lines indicate the optimal adsorption energies for hydrogen storage by physisorption. Reproduced from reference [122].

Some studies explored the influence of the concentration of doping elements on the hydrogen-storage properties in doped graphene [103,104,109,112,122]. For instance, DFT calculations and molecular dynamics were used to study H_2_ adsorption on Li-doped graphene (C_17_Li and C_7_Li). At atmospheric pressure and 300 K, the C_7_Li composite could store up to 6.2 wt.% hydrogen, with an adsorption energy of −0.19 eV/H_2_ [109]. Interestingly, this material satisfies the DOE requirements. Therefore, it can be a promising material for hydrogen storage. In another study, hydrogen storage on Ti- and Ti_2_-doped graphene was investigated using the PBE functional, as shown in Figure 5 [122]. Ti_2_-doped graphene was found to be a better material for hydrogen storage than Ti-doped graphene (Figure 5d). Thus, these studies show that the concentration of doping elements plays an important role in determining the hydrogen storage capacity of doped graphene [103,104,109,112,122].

### 4.3. Hydrogen Storage on Cluster-Doped Graphene

**Figure 6:**
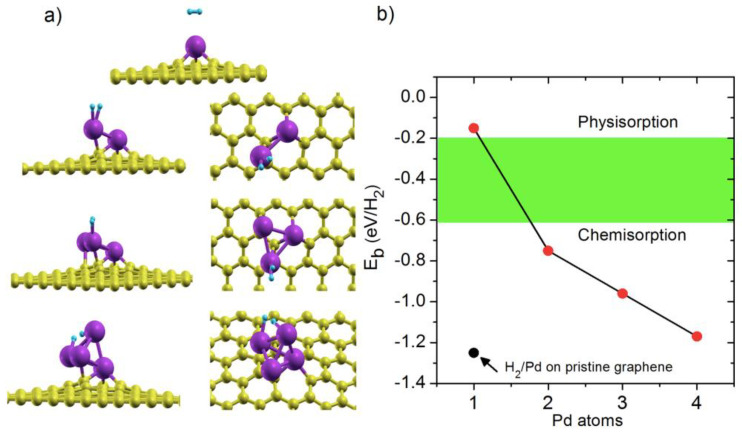
(**a**) Relaxed structures and (**b**) variation of the adsorption energies for a single H_2_ on the graphene-supported Pd_n_ (n = 1−4) clusters as a function of cluster size. The adsorption energy for a single H_2_ on a Pd atom deposited on a pristine graphene is also given. The energy ranges for chemical and physical H_2_ adsorption are marked. Yellow, purple, and cyan circles represent carbon, palladium, and hydrogen atoms, respectively. The optimum energy range for reversible H_2_ absorption/desorption is marked in a green rectangle. Reproduced with permission from reference [115].

Hydrogen storage on cluster-doped graphene has been explored [113,115,116,117,120]. For example, Ti_4_- and Ni_4_-doped graphene structures were studied for hydrogen storage using PBE functional [113]. It was observed that the Ti_4_-doped graphene has a better gravimetric capacity (3.4 wt.%) than Ni_4_-doped graphene (0.30 wt.%). In another study, H_2_ storage on Pd_6_-doped graphene was examined by using the PW91 functional. It was demonstrated that Pd_6_-doped graphene is a good material for hydrogen storage [117]. In another study, hydrogen storage was computed on Pd_n_-doped graphene (n = 1–4) by using the PBE functional [115]. The variation of the H_2_ adsorption energies on the Pd_n_ (n = 1–4) clusters-doped graphene supported as a function of cluster size is illustrated in Figure 6. The single H_2_ adsorption energy increases as the Pd cluster size increases. Also, Pd_4_-doped graphene can adsorb four molecules of H_2_ while satisfying the requirements of the DOE, making it a good candidate for hydrogen storage.

### 4.4. Hydrogen Storage on Co-Doped Graphene

**Figure 7:**
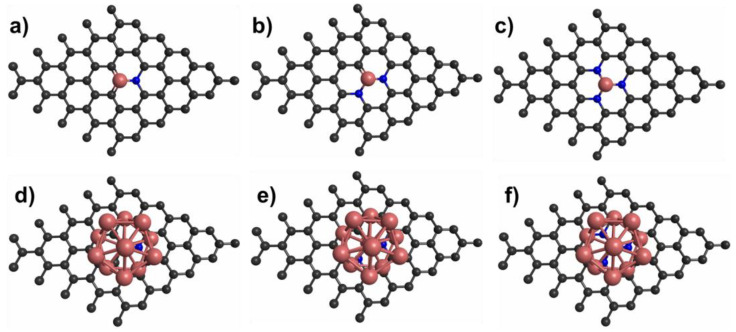
(**a**–**c**) Models of single-atom co-doped graphene; (**d**–**f**) models of cluster and single-atom co-doped graphene.

Hydrogen storage on co-doped graphene has been investigated by various studies [119,125,126,127,128,129,130,131]. In this case, two types of atoms are embedded in the graphene structure. Figure 7 shows the different configurations that have been explored. Numerous co-doped graphene systems, such as B–Pd [119], B–Li [125], 3N–Li [126,128], 3N–Ti [127], 3N–Pd, 3N–Pd_2_, 3N–Pd_3_, 3N–Pd_4_ [129], N–Sc, 2N–Sc, 3N–Sc [130], N–Cu, 2N–Cu, and 3N–Cu [131], have been explored for hydrogen storage. Interestingly, most of these systems meet the DOE requirement for hydrogen storage via physisorption [119,125,126,127,128,129,130,131]. For instance, hydrogen adsorption on B-Li co-doped graphene structure was studied using the PBE functional [125]. It was computed that B-Li co-doped graphene can adsorb up to three H_2_ molecules with an adsorption energy of −0.19 eV/H_2_. Also, the hydrogen storage properties for Ti-3N co-doped graphene structure were computed using the PBE functional considering the van der Waals interactions [127]. The study demonstrated the ability of Ti-3N co-doped graphene to adsorb up to three H_2_ molecules with the adsorption energy required by the DOE [127]. In another investigation, hydrogen storage properties for Sc-N, Sc-2N, and Sc-3N co-doped graphene were studied using the PBE functional considering the van der Waals interactions [130]. The average adsorption energies of H_2_ molecules on Sc-N, Sc-2N, and Sc-3N co-doped graphene structures are reported in Table 1. The results show that the H_2_ adsorption energy on co-doped graphene increases gradually as the N concentration increases. In terms of gravimetric capacity, N–Sc, 2N–Sc, and 3N–Sc co-doped graphene can adsorb up to six H_2_ molecules with adsorption energies of −0.15, −0.17, and −0.19 eV, respectively; see Table 1 [130]. Also, DFT-based theoretical computations were conducted for studying the H_2_ adsorption on Cu-N, Cu-2N, and Cu-3N co-doped graphene structures employing the B3LYP functional [31]. It is observed that the Cu-3N co-doped graphene structure is the best candidate for hydrogen storage. These results show that co-doped graphene structures are promising candidates for hydrogen storage.

## 5. Hydrogen Storage on Graphene with Vacancies

**Figure 8:**
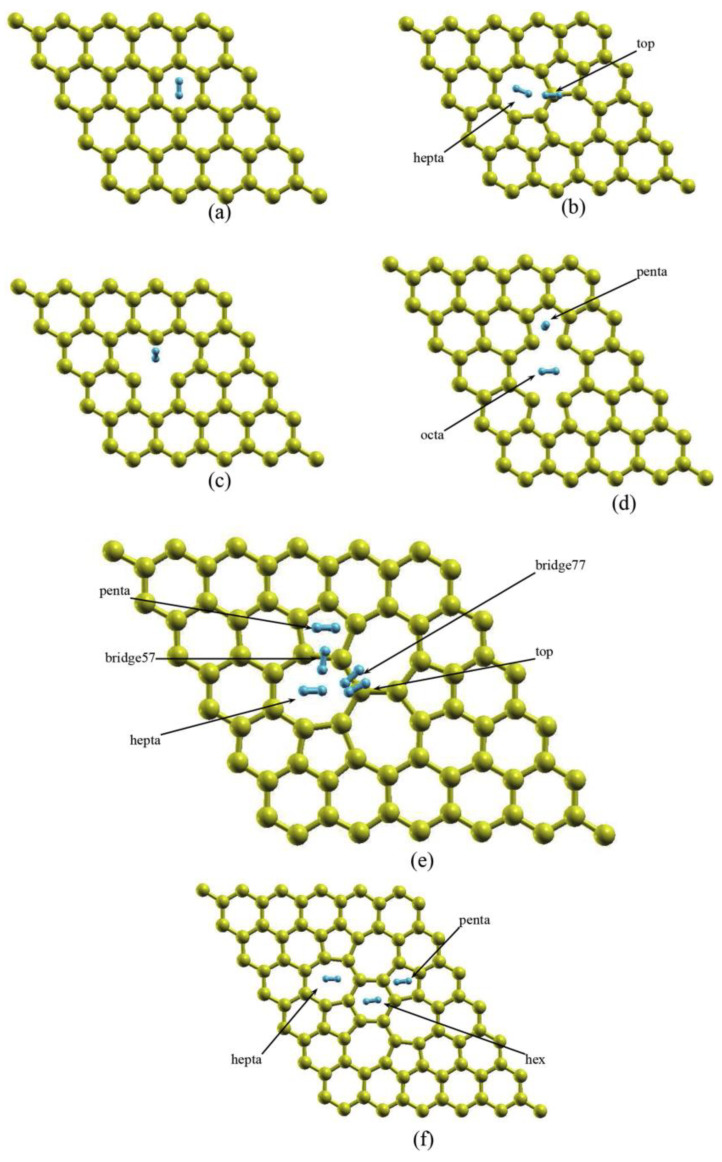
Supercells for hydrogen binding over individual defect systems depicting different initial positions for the adsorption of a hydrogen molecule: (**a**) pristine, (**b**) Stone–Wales, (**c**) single vacancy, (**d**) double vacancy 585, (**e**) double vacancy 555–777, and (**f**) double vacancy 5555–6–7777. Reproduced with permission from reference [134].

Graphene with vacancies exhibits better reactivity than pristine graphene [132,133]. Theoretical studies have shown that different defects can be introduced in the graphene structure to improve its hydrogen storage properties [122,134,135,136]. For instance, hydrogen storage on different types of vacancies such as Stone–Wales (SW), single vacancy (SV), and three types of double vacancy was theoretically studied; see Figure 8 [134]. Graphene with SV and mixed SW–SV had gravimetric densities of 5.81 and 7.02 wt.%, respectively, for hydrogen storage [134]. A recent study demonstrated hydrogen storage in double-vacancy graphene (DVG) by using the PBE functional [122]. This structure could store up to nine H_2_ molecules (Figure 5d). These results show that graphene structures with vacancies are good candidates for hydrogen storage.

## 6. Hydrogen Storage on Doped-Decorated Graphene

**Figure 9:**
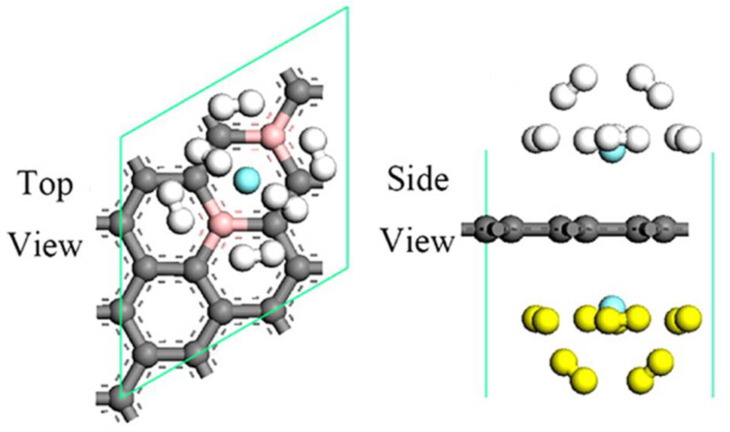
Optimized structures of twelve hydrogen molecules’ adsorption on Y coated double-sided graphene with boron doping. Cyan, pink, and dark gray spheres denote Y, boron, and carbon atoms. Light gray and yellow spheres are hydrogen molecules attaching on the top and opposite sides of graphene, respectively. Reproduced with permission from reference [140].

So far, different doped-decorated graphene systems have been studied for hydrogen storage with promising results [54,55,60,91,93,107,126,128,137,138,139,140,141,142,143,144,145]. In this approach, the doping atoms are embedded in the graphene structure, while the decorating atoms are deposited on the doped graphene sheet. For instance, the use of Mg-decorated B-doped graphene for hydrogen storage was examined by using local-density approximation (LDA) methods [137]. The adsorption of six H_2_ molecules on a Mg-decorated B-doped graphene corresponds to a computed adsorption energy of −0.55 eV/H_2_, making this material a good candidate for hydrogen storage. In another study, hydrogen adsorption on Ni-, Pd-, and Co-decorated B-doped (BC_5_) graphene was investigated using the PW91 functional [139]. When 11 H_2_ molecules were adsorbed on Ni-decorated B-doped graphene, the calculated adsorption energy was −0.34 eV/H_2_. Recently, hydrogen adsorption on graphene doped with two B atoms and decorated with two Y atoms was investigated employing the Perdew–Wang (PW) functional; see Figure 9 [140]. This system could store 12 H_2_ molecules with an adsorption energy of −0.568 eV/H_2_. In addition, metal-decorated B-doped (BC_5_) graphene was studied for hydrogen storage using the PW91 functional [141]. Up to nine H_2_ molecules could be adsorbed on Ni- and Ti-decorated B-doped graphene with an adsorption energy of −0.43 and −0.41 eV/H_2_, respectively. Another study examined the use of La-decorated B-doped graphene for hydrogen storage by using the LDA method [142] and showed that up to six H_2_ molecules were adsorbed with an adsorption energy of −0.53 eV/H_2_. These studies show that decorated-doped graphene systems are good candidates for hydrogen storage.

## 7. Hydrogen Storage on Graphene with Vacancy-Doping

**Figure 10:**
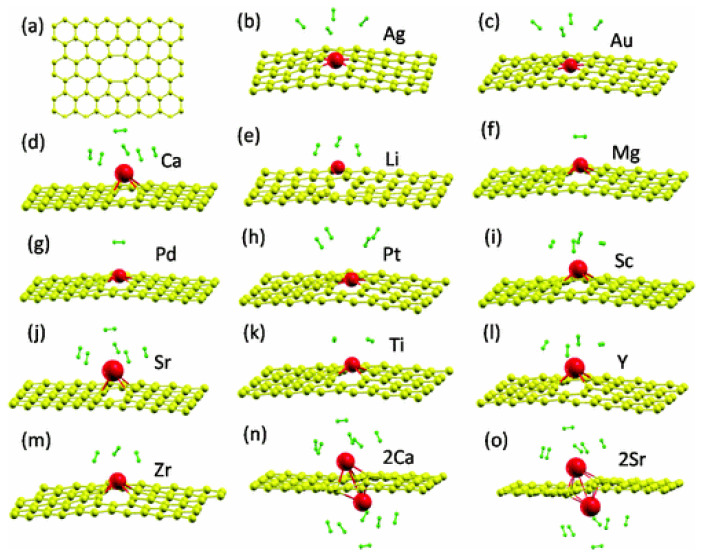
The relaxed atomic geometries for (**a**) a graphene sheet with a DCV, (**b**–**m**) the 12 different metal adatoms with their maximum hydrogen capacity, and (**n**,**o**) the Ca and Sr systems with adatoms adsorbed on both sides of the DCV at their maximum H_2_ capacities. Reproduced with permission from reference [148].

Embedding vacancies-dopants in the graphene structure is another strategy to improve the reactivity of graphene for hydrogen storage. Various doped graphene structures with vacancies have been examined [68,69,93,119,125,143,146,147,148,149,150,151,152,153,154,155,156]. For instance, graphene with SW defects and doped with Li was investigated using a PBE functional with dispersion corrections [68]. This structure can adsorb four H_2_ molecules with an optimal adsorption energy for hydrogen storage. In another study, hydrogen adsorption on graphene with double vacancies and doped with Li was investigated using a PBE functional with dispersion correction [125]. This system adsorbed three H_2_ molecules with an adsorption energy of −0.20 eV/H_2_. Hydrogen storage using DVG and doped with Ti was studied using the PBE approximation [146]. An adsorption energy of −0.21 eV/H_2_ was computed for four H_2_ molecules on each side of this structure. Recently, DVG (555–777) doped with a Pd_4_ cluster was studied using the PBE functional [147]. An adsorption energy of −0.64 eV/H_2_ was calculated when five H_2_ molecules were adsorbed on this system. In another study, hydrogen storage in DVG doped with 12 metals (Ag, Au, Ca, Li, Mg, Pd, Pt, Sc, Sr, Ti, Y, and Zr) was studied by using the generalized gradient approximation; see Figure 10 [148]. Computations showed that Ca and Sr have the largest capacity and can store up to six H_2_ molecules each. More recently, hydrogen storage in graphene structures with double vacancies (585 and 555–777) doped with Ca was studied using the PBE functional with dispersion corrections [149]. These structures can store up to six H_2_ molecules each. Also, the capacity of DVG doped with Li for hydrogen storage was computed by using the PW functional [69]. The storage capacity of this structure was 7.26 wt.% when Li was doped on both sides of the defective graphene. These investigations show that graphene structures with vacancy-doping are good candidates for hydrogen storage.

## 8. Hydrogen Storage on Graphene with Co-Doping and Vacancies

Adding co-doping and vacancies in the graphene structure is another strategy used to improve the reactivity of graphene for hydrogen storage. To date, several modified graphene structures with co-doping and vacancies have been studied for hydrogen storage [105,119,125,126,128,129,152,153,156]. For instance, Li-B co-doped DVG was studied for hydrogen storage using the PBE functional [125]. This structure can adsorb three H_2_ molecules with an adsorption energy like that required by the DOE. In another study, Li-doped pyrrolic N-doped graphene was studied for hydrogen storage employing the PBE functional and considering the van der Waals corrections [126]. This structure can adsorb three H_2_ molecules with an adsorption energy of −0.18 eV/H_2_. Also, different porphyrin-doped graphene structures were studied for hydrogen storage using the PBE functional [105]. It was computed that Sc-, Ti-, and V-porphyrin-doped graphene can be good candidates for hydrogen storage, since these structures meet the requirements established by the DOE. Recently, Be-porphyrin-doped graphene structure was computed for hydrogen storage employing the PW functional [152]. According to the adsorption energy established by the DOE, a maximum of four H_2_ molecules can be adsorbed on Be-porphyrin-doped graphene. These studies show that graphene structures with co-doping and vacancies are good candidates for hydrogen storage via physisorption.

## 9. Conclusions and Future Directions

This review analyzed the advances in the design of novel graphene-based hydrogen storage materials, highlighting the modifications made to the graphene structure based on DFT studies to improve its hydrogen storage properties. To date, various modified graphene structures, such as decorated graphene, doped graphene, graphene with vacancies, graphene with vacancies-doping, as well as decorated-doped graphene, have been explored to modify the reactivity of pristine graphene. Most of these modified graphene structures are good candidates for hydrogen storage. From this detailed review, the following conclusions and future directions can be suggested:

(a) Graphene structures decorated with single-atoms or atom clusters for hydrogen storage have been examined. The commonly used strategy is to decorate graphene with single atoms. Therefore, more studies on cluster-decorated graphene for hydrogen storage are required. Further, since bimetallic and trimetallic systems are known to have properties very different from those of monometallic systems, it will be interesting to investigate graphene decorated with bimetallic or trimetallic clusters for hydrogen storage. Most graphene systems decorated with clusters or atoms comply with the DOE requirement for hydrogen storage via physisorption. Furthermore, several of the investigated materials, in particular, graphene decorated with Al, Ca, Li, and Ti, had gravimetric capacities higher than the target set by the DOE.
(b) The use of doped graphene for hydrogen storage has been widely investigated. Several strategies, such as single-atom doping, cluster doping, and co-doping, were implemented. These types of doping substantially modify the reactivity of graphene, providing promising materials for hydrogen storage. However, theoretical studies on cluster-doped and co-doped graphene for hydrogen storage are still scarce. Therefore, it is necessary to conduct more detailed research on cluster-doped and co-doped graphene for hydrogen storage.
(c) The use of graphene with vacancies, doped-decorated graphene, and graphene with vacancies-doping are other strategies to modify the reactivity of pristine graphene for hydrogen storage. The existing studies have shown promising results for hydrogen storage. However, comprehensive studies on these systems are necessary.
(d) The graphene structures with co-doping and vacancies have been examined for hydrogen storage. The available studies show that graphene structures with co-doping and vacancies are good candidates for hydrogen storage. However, more studies are required on this type of modified graphene.
(e) Future theoretical studies on modified graphene for hydrogen storage must adopt dispersion corrections. Many existing studies did not include these corrections, limiting the quality of the results. Future studies should also report the gravimetric capacity of the systems as it is an important parameter to determine whether a material is a good candidate for hydrogen storage. Many existing studies only reported the adsorption energy of the H_2_ molecule, which is not enough to identify new materials for hydrogen storage.
(f) These theoretical results discussed herein should motivate experimental groups to experimentally validate the theoretical predictions, as many modified graphene systems are shown to be good candidates for hydrogen storage. The knowledge of these systems can be systematized, and the systems can be experimentally evaluated for hydrogen storage.

## Figures and Tables

**Table 1:** The calculated average adsorption energies (eV/H_2_) of H_2_ molecules on Sc-decorated N-, 2N-, 3N-doped graphene structures with 1–6 H_2_ molecules adsorbed. Reproduced with permission from reference [130].

Number of H_2_	1	2	3	4	5	6
Sc-decorated N-doped graphene	0.19	0.18	0.18	0.18	0.16	0.15
Sc-decorated 2N-doped graphene	0.25	0.23	0.22	0.20	0.18	0.17
Sc-decorated 3N-doped graphene	0.34	0.32	0.29	0.27	0.23	0.19

## Data Availability

Data are contained within the article.
